# Transmission Electron Microscopy Confirmation of *Orientia tsutsugamushi* in Human Bile

**DOI:** 10.3201/eid2612.202188

**Published:** 2020-12

**Authors:** Yujeong Lee, Seung Il Kim, Yoon-sun Yi, Hayoung Lee, Joo-Hee Hwang, Edmond Changkyun Park, Sangmi Jun, Chang-Seop Lee

**Affiliations:** Korea Basic Science Institute, Cheongju, South Korea (Y. Lee, S.I. Kim, Y.-s. Yi, H. Lee, E.C. Park, S. Jun);; Korea Research Institute of Chemical Technology, Daejeon, South Korea (Y. Lee, S.I. Kim, E.C. Park, S. Jun);; University of Science and Technology, Daejeon (S.I. Kim, H. Lee, E.C. Park);; Jeonbuk National University, Jeonju, South Korea (J.-H. Hwang, C.-S. Lee);; Jeonbuk National University Hospital, Jeonju (J.-H. Hwang, C.-S. Lee)

**Keywords:** Orientia tsutsugamushi, transmission electron microscopy, human bile, eschar, PCR, South Korea, vector-borne infections, scrub typhus, bacteria, coccobacillus

## Abstract

Scrub typhus, the third most frequently reported infectious disease in South Korea, causes serious public health problems. In 2019, we collected a bile specimen from a patient with scrub typhus through percutaneous transhepatic gallbladder drainage and performed transmission electron microscopy to confirm the ultrastructure of *Orientia* *tsutsugamushi*.

*Orientia tsutsugamushi* is a gram-negative obligately intracellular coccobacillus and a causative pathogen of scrub typhus, which is transmitted to humans by bites from trombiculid (chigger) mites (*1*). Scrub typhus is a prevalent acute febrile disease that mainly occurs in the Asia-Pacific region, infecting »1 million persons worldwide each year. In South Korea, infections have increased rapidly since 2014 because of climate change, increased outdoor activities, and numbers of elderly farmers (*2*). The most typical clinical manifestation of scrub typhus is an eschar at the site of the bite (Figure, panel A); symptoms include fever, headache, muscle pain, nausea, and vomiting (*3*). Without proper diagnosis and antimicrobial drug treatment, severe illness with multiple organ system involvement can occur; the death rate is »10% (*4*). Immunohistochemical staining for *O. tsutsugamushi* antigens have revealed extensive endothelial cell infection in the heart, lung, kidney, pancreas, skin, and brain (*5*). Bacteria also have been detected in cardiac muscle cells and in macrophages in the liver and spleen (*5*,*6*).

In humans, the liver secretes »1 L of bile daily into the intestinal tract. However, little information is available about the presence of gram-negative bacteria in bile (*7*). Pathogenic microorganisms must endure potential impediments, such as variations in pH, low oxygen levels, nutrient limitation, and elevated osmolarity, to survive in this harsh environment (*7*). We collected bile from a patient with scrub typhus in South Korea (Figure, panel B) and visualized the ultrastructure of *O. tsutsugamushi* in the clinical sample using transmission electron microscopy.

In 2019, a 68-year-old woman reported fever, drowsy mental state, abdominal pain, and reduced oral intake. These symptoms had begun 7 days earlier. Her vital signs were blood pressure 100/60 mm Hg and body temperature 38.9°C. Laboratory analysis revealed a leukocyte count 10,350/mL (reference range 4,800–10,800/mL), platelet count 45,000/mL (reference 130,000–450,000/mL), serum creatinine 0.4 mg/dL (reference 0.7–1.7 mg/dL), aspartate aminotransferase 31 IU/L (reference 12–33 IU/L), alanine aminotransferase 41 IU/L (reference 5–35 IU/L), total bilirubin 1.72 mg/dL (reference 0.2–1.2 mg/dL), and C-reactive protein 196.86 mg/L (reference <5 mg/L). Abdominal computed tomography scan detected acute cholecystitis, and percutaneous transhepatic gallbladder drainage was performed. The presence of acute cholecystitis in scrub typhus cases is rare (5 [1.1%] instances of 442 cases) (*8*). *O. tsutsugamushi* can also cause liver injury in some patients, but the patient reported here did not have any such signs (*9*). We confirmed scrub typhus using indirect immunofluorescence assay (IgG 5,120) and nested PCR selective for the 56-kDa gene of *O. tsutsugamushi* (Appendix). The *O. tsutsugamushi* identified belonged to the Boryong strain (the most common strain in South Korea). We also detected an eschar in the right inguinal area (Figure, panel A). The patient completely recovered after doxycycline treatment.

**Figure:**
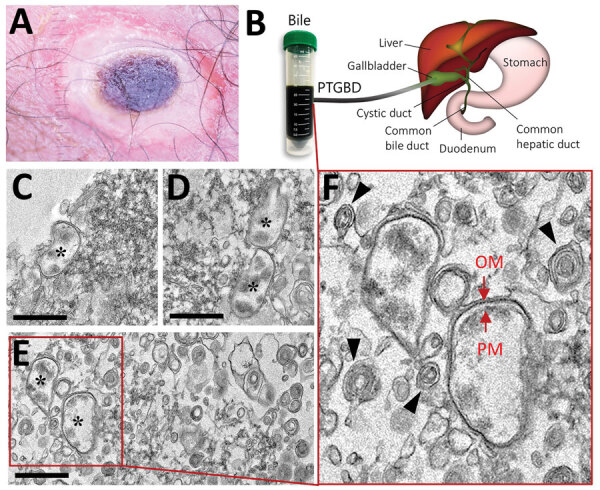
Findings from a 68-year-old woman with scrub typhus, South Korea, 2019. A) Eschar in the right inguinal area. B) Human bile collected through percutaneous transhepatic gallbladder drainage in the gallbladder of a patient affected with scrub typhus. C–F) Transmission electron microscopy images of *Orientia* *tsutsugamushi* in the bile. Bacteria (black asterisks); outer membrane (OM) and plasma membrane (PM) (red arrows); multilamellar body (black arrowheads); Scale bars indicate 1 μm.

A drainage tube was placed in the patient’s gallbladder, and the bile was directly discharged and collected through the tube (Figure, panel B). We tested the bile specimen for pathogens using nested quantitative reverse transcription PCR and DNA sequencing to detect a specific *O. tsutsugamushi* gene encoding a 56-kDa protein (Appendix) (*10*). After chemical fixation, the sample was embedded in 100% Epon 812 resin and ultrathin (»80-nm thick) sections were stained with 2% uranyl acetate and 1% lead citrate (Appendix) (*10*). This sample preparation method might not preserve the ultrastructure of live bacteria, but structural features of the bacteria can be clearly observed. The ultrastructural details were acquired using transmission electron microscopy at 120 kV. Despite the presence of a wide variety of components, we detected *O. tsutsugamushi* in the bile (asterisks in Figure, panels C–E). The bacteria showed a coccobacillus shape and were 0.5–0.7-μm in diameter and 1.2–2.5-μm long, all typical features of *O. tsutsugamushi* (*5*,*10*). The bacterial cytoplasm was surrounded by an outer membrane, an internal plasma membrane, and a peptidoglycan layer (Figure, panel F). Moreover, the periplasmic space appeared as an electron-lucent gap between the 2 membranes. We also observed a thicker outer leaflet of the cell wall membrane, which is a typical and diagnostic sign of *Orientia* (*5*). We also detected multilamellar bodies, which are cholesterol-carrying particles, in the bile sample (black arrowheads in Figure, panel F).

Previously, human scrub typhus disease was studied using a mouse model mimicking the disease and examining clinical samples postmortem (*5*,*6*). However, the host cell of *O. tsutsugamushi* in humans has not been completely defined. In this study, we confirmed detection of *O. tsutsugamushi* in human bile, an environment in which bacterial survival is challenging. This observation (i.e., the presence of *O. tsutsugamushi* in human bile) might be useful for diagnosing scrub typhus in patients who do not show clear eschars or skin rash, broadening the potential routes for diagnosing the disease.

Appendix Additional information for transmission electron microscopy confirmation of *Orientia tsutsugamushi* in human bile.
