# Supplementary material for: Transmission Electron Microscopy Confirmation of Orientia tsutsugamushi in Human Bile
Source: Emerg Infect Dis. 2020 Dec;26(12):3101–3. doi: 10.3201/eid2612.202188 (PMC7706943; doi:10.3201/eid2612.202188)
Supplement: Appendix — Additional information for transmission electron microscopy confirmation of Orientia tsutsugamushi in human bile. [file 20-2188-Techapp-s1.pdf]

Article DOI: https://doi.org/10.3201/eid2612.202188

Transmission Electron Microscopy Confirmation of *Orientia tsutsugamushi* in Human Bile

Appendix

Genotyping by DNA Amplification and Sequencing

Peripheral blood mononuclear cells collected from acute-phase blood samples of patients with scrub typhus were purified using a QIAamp DNA Blood Mini Kit (QIAGEN, Hilden, Germany) according to the manufacturer's protocol. Nested PCR was performed. Primers 34 (forward, 5′-TCA AGC TTA TTG CTA GTG CAA TGT CTGC-3′; the 56-kDa gene based on the Gilliam strain) and 55 (5′-AGG GAT CCC TGC TGT GCT TGC TGCG-3′) were used in the first PCR. Nested PCR primers 10 (5′-GAT CAA GCT TCC TCA GCC TAC TAT AAT GCC-3′) and 11 (5′-CTA GGG ATC CCG ACA GAT GCA CTA TTA GGC-3′) were used in the second PCR amplification to generate a 483-bp fragment. Nested PCR was performed as described previously by Kim et al. (*1*). The amplified PCR products were confirmed using 1.2% agarose gel electrophoresis, purified with a QIAquick gel extraction kit (QIAGEN) and sent to COSMO Genetech (Seoul, South Korea) for sequencing.

Identification of 56-kDa Gene by DNA Amplification and Sequencing Using Bile-Derived DNA

Total genomic DNA was extracted from the bile using a QIAamp DNA Mini Kit (QIAGEN), as previously described (*2*,*3*). The 56-kDa gene of *O. tsutsugamushi* was amplified using nested PCR, as previously described (*4*). Briefly, initial rounds of amplifications were performed using touchdown PCR, and products from the initial amplification were used as the template in the second PCR reaction to generate an 83-bp fragment. Nested PCR was performed using Solg 2X Multiplex PCR Smart mix (Solgent, Daejeon, South Korea). Levels of the 56-kDa gene were normalized using the human Gapdh (h*Gapdh*) gene. Information on the primers used in this study is summarized in the Appendix Table. The final PCR products were visualized with a chemiDoc XRS+ system (Bio-Rad,

Hercules, CA, USA) (Appendix Figure 1) and sent to COSMO Genetech for sequencing. The sequencing results were aligned with the original sequence of the 56-kDa gene using Clustal Omega, which revealed the 56-kDa gene in bile-derived DNA (Appendix Figure 2).

Transmission Election Microscopy (TEM)

For ultrastructural analysis of ultrathin sections, collected human bile was pelleted via centrifugation and resuspended in 0.1% glutaraldehyde solution and 4% paraformaldehyde, 3.5% sucrose in phosphate buffer (0.1 M, pH 7.4) for 2 h at 4°C. The samples were washed in phosphate buffer, and then post-fixed in 1% osmium tetroxide (Electron Microscopy Sciences, Hatfield, PA, USA) in phosphate buffer (0.1 M, pH 7.4) for 2 h at 4°C. Samples were dehydrated in a graded ethanol series, followed by propylene oxide, and then progressively infiltrated with a 2:1, 1:1, and 1:2 mixture of propylene oxide and Epon 812 resin (Electron Microscopy Sciences). Samples finally embedded in 100% Epon 812 resin and polymerized at 70°C for 24 h. Ultrathin plastic sections (80-nm thick) were cut at room temperature using a Leica EM UC6 ultramicrotome (Leica Microsystems GmbH, Wetzlar, Germany) and collected on 200-mesh carbon-coated grids. The grids were post-stained with 2% aqueous uranyl acetate and 1% lead citrate at room temperature for 15 and 5 min, respectively. A FEI Tecnai G2 Spirit Twin 120 KV TEM (FEI Company, Hillsboro, OR, USA) was used for TEM analysis (*4*,*5*)

References

1. Lee YM, Kim DM, Lee SH, Jang MS, Neupane GP. Phylogenetic analysis of the 56 kDa protein genes of *Orientia tsutsugamushi* in southwest area of Korea. Am J Trop Med Hyg. 2011;84:250–4. PubMed https://doi.org/10.4269/ajtmh.2011.09-0601

2. Neri V, Margiotta M, de Francesco V, Ambrosi A, Valle ND, Fersini A, et al. DNA sequences and proteic antigens of *H. pylori* in cholecystic bile and tissue of patients with gallstones. Aliment Pharmacol Ther. 2005;22:715–20. PubMed https://doi.org/10.1111/j.1365-2036.2005.02644.x

3. Lee JW, Lee DH, Lee JI, Jeong S, Kwon KS, Kim HG, et al. Identification of *Helicobacter pylori* in gallstone, bile, and other hepatobiliary tissues of patients with cholecystitis. Gut Liver. 2010;4:60–7. PubMed https://doi.org/10.5009/gnl.2010.4.1.60

4. Ro HJ, Lee H, Park EC, Lee CS, Il Kim S, Jun S. Ultrastructural visualization of *Orientia tsutsugamushi* in biopsied eschars and monocytes from scrub typhus patients in South Korea. Sci Rep. 2018;8:17373. PubMed https://doi.org/10.1038/s41598-018-35775-9

5. Goldsmith CS, Miller SE. Modern uses of electron microscopy for detection of viruses. Clin Microbiol Rev. 2009;22:552–63. PubMed https://doi.org/10.1128/CMR.00027-09

Appendix Table. List of primers used in the study

Primer name	Sequence
c56k F	5′-AGCTGATCGTGACTTTGGGATT-3′
c56k R	5′-AGCATTTGATAATGCAGCAAGACC-3′
rt56k F	5′-CCTAACATACCTCAGGCGCA-3′
rt56k R	5′-AACCAAGCGATCCTAGCTGC-3′
Human GAPDH F	5′-CGGGAAACTGTGGCGTGATG-3′
Human GAPDH R	5′-ATGACCTTGCCCACAGCCTT-3′

*F, forward; R, reverse.

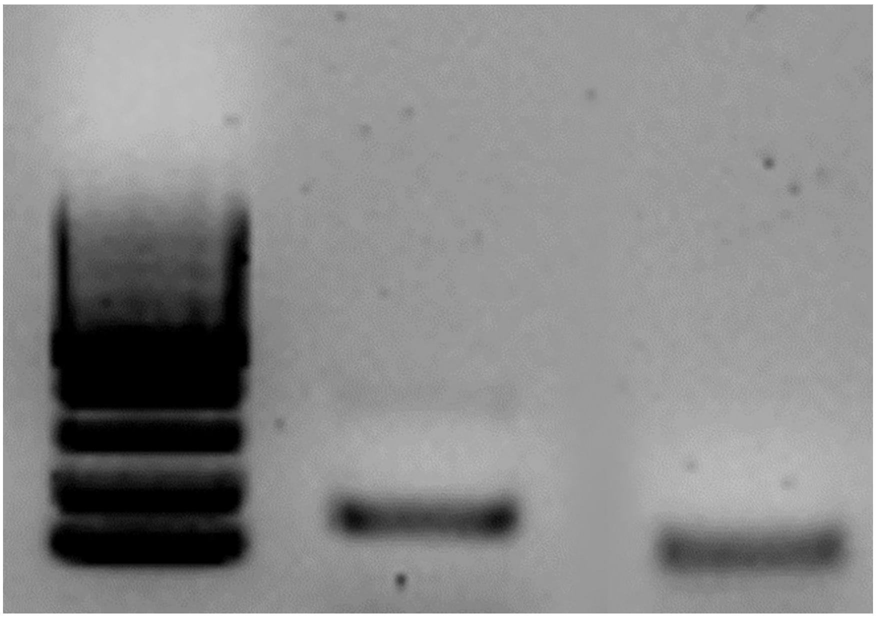

Appendix Figure 1. PCR results in bile-derived DNA of *Orientia tsutsugamushi*. Each lane indicates the ladder and the amplification product obtained using primer pairs for *hGapdh* and *56 kDa*, respectively

```
CLUSTAL O(1.2.4) multiple sequence alignment


PCR_product      CCTAACATACCTCAGGCGCAAGCACAAGCGGCGCGGCCTCAGCTAATATGATGAGCACGT    60
56kDa            CCTAACATACCTCAGGCGCAAGCGCAAGCTGCACAGCCTCCGCTTAATGATCAGAAGCGT    60
                 *********************** ***** ** * ***** *** *             ***

PCR_product      GCTGCAGCTAGGATCGCTTGGTTA        84
56kDa            GCTGCAGCTAGGATCGCTTGGTT-        83
                 ***********************
```

Appendix Figure 2. Sequence alignment for *Orientia tsutsugamushi*.
